# The Water Cure for Hydropathy, from the British and Foreign Medical Review

**Published:** 1847-01

**Authors:** 


Art. VIII.—*The Water Cure, or Hydropathy.* From the British and Foreign Medical Review. By John Forbes, M.D, F.R.S., one of the Editors of the "Cyclopædia of Practical Medicine," Editor of the "British and Foreign Medical Review," etc., etc. Philadelphia: Lindsay & Blakiston. 1847.

A popular modern writer has termed the soul "a succedaneum for salt," without some portion of which the body would inevitably putrefy. Some admixture of truth is equally essential to every heresy, whether in politics, medicine, or religion. A palpable, bare-faced absurdity, unrelieved by any gleams of reason, would not long pass current in any enlightened community. Where, therefore, we find any doctrine or practice prevailing for a length of time, and over a wide extent of country in which men read and think, we may take it for granted that it has some show of truth about it. It has some foundation, or it would soon fall to the ground. Men do not believe without evidence, though the evidence is often most insufficient, and the opinions formed most false. Homœopathy would be voted by any man of sense, hearing its tenets explained for the first time, a monstrous absurdity, and yet thousands are duped by its pretensions every day. Why is it? It is because remarkable recoveries sometimes take place while these doctors are standing by, and their infinitesimal doses get the credit of the cure. If there were no *vis medicatrix naturæ*, homœopathy would soon come to an end. But the homœopaths do just nothing; they leave the curative powers of nature to themselves, and these oftentimes triumph over disease. Recoveries are not uncommon under the homœopathic treatment, and homœopathy has the seeming of reality about it.

Hydropathy, within a few years, has been added to the list of humbugs, and is now attracting to it the attention of men in all countries where medicine is pursued as a separate art. We are not sure that, at present, it is not the prevailing superstition of the times. In some essential respects it differs from its kindred system; and while, like homœopathy, as a system it is most absurd, like it, there is something about it, if not truthful, that bears a strong resemblance to the truth. Cold water, all physicians know, is a most powerful remedial agent, "and it may turn out that its application to the body is capable of being beneficially applied, in the cure of diseases, in modes of greater efficacy, and to a much greater extent, than has hitherto been practiced by medical men." If it should so turn out, and we are prepared to believe it will, there remains only one course for the members of the profession to pursue, and that is, as expressed by Dr. Forbes, "to adopt the improvements, if such they are, regardless of their origin, or their past or present relations." We see no reason to question that cold water is susceptible of far wider and more efficient applications than have yet been made of it in the management of disease, and we would not be frightened out of a fair appreciation of its virtues because an ignorant pretender has erected upon its cures a ridiculous system. Let us welcome truth wherever we can find it, and though a savage, or to suppose a stronger case, a *quack* might be the author of it, we should feel constrained to give him credit for his discovery, and thank him too. We must not forget to what purely fortuitous circumstances we are indebted for some of our most esteemed medicines; nor are we to leave valuable agents in the hands of empirics because they may have had most to do in drawing attention to them. On this subject we adopt the sentiments of Dr. Forbes, who discourses thus concerning it:

When the religious reformer proposed to adapt profane airs to church psalmody, saying that he saw no good reason why the devil should have all the good tunes to himself, he is generally supposed to have acted as wisely as he thought shrewdly and spoke quaintly. In like manner, we see no good reason why the doctors of the orthodox or legitimate school, should refuse to accept good things, even at the hand of the hydropathists. They have done like things before now, as the pharmacopœia, in more pages than one, can testify; and we have not heard that there has been any great reason for regretting that they did so. For our own parts, we avow ourselves of such a catholic spirit, and so lowly minded withal, as to be ready to grasp any proffered good in the way of HEALING, whosoever may be the offerers, and wheresoever they may have found it. Not merely hydropathy, but even mesmerism, yea, stark-naked and rampant quackery itself, may, in this sense, be a welcome knocker at the gate of physic. It is not the demerits of the donor or the birthplace of the gift, that, in such a case, we are bound to look to—but simply whether it is qualified to aid us in our glorious and divine mission of soothing the pains of our fellow-men. If it is so qualified, the baseness of its source will be lost in the glory of its use; and, if ought of its original impurity still attaches to its application in our hands, the fault will be in us, not in it. A saint may sing the devil's tunes without contamination; a hero may wield the weapon he has wrested from a robber or a murderer; the medicament or the formula of the most arrant quack may be hallowed in the prescription of the true physician.

Perhaps our readers have not met anywhere with a personal description of Preissnitz, the founder of Hydropathy. Sir Charles Scudamore thus describes him:

His countenance is full of self-possession; rather agreeable; mild, but firm in expression; with an eye of sense, and a pleasing smile. The small-pox and the loss of some front teeth from an accident, impair his good looks. His manners are sufficiently well-bred. On closer acquaintance, you discover that he is quick in perception; is reflective; prompt, however, in decision; simple and clear. He inspires his patients with the most entire confidence, and he exacts implicit obedience.

Other travelers give a similar description of him.

They all agree in stating that he is a most arbitrary and tyrannical despot, issuing laws as irrevocable as those of the Medes and Persians, commanding obedience with a haughtiness that might well excite admiration and envy even in an autocrat, and exciting as much fear in his patients as is found in the subjects of the Grand Turk himself. He is also represented as remarkably cool and collected in emergencies, ever ready with his remedy on occasions of danger, and possessed of an imperturbable self-reliance. These traits suffice to prove that he is a man of original and powerful mind, exactly adapted to carry out a novel and startling practice. While his firm and decided manner is calculated to secure the confidence of his patients, his coolness and self-reliance enable him easily to bear the responsibility by which such confidence is attended.

His practice originated in a succession of trifling accidents, by which he was led to employ bathing in a neighboring spring for the relief of disease. It is not necessary to give them in detail. Success in the first attempts procured him a local renown, and he became the village doctor. From villagers his fame soon spread to patients of a higher rank, and Graeffenberg gradually became the resort of the hipped and the halt from all the surrounding district. By these his praises were sung louder and louder, until all the world began to furnish him patients by the hundred. He now possesses an enormous establishment, capable of containing several hundreds of patients, which is almost constantly crowded with ladies and gentlemen of every degree, and from every nation; while his disciples and followers, as is well known, have spread themselves throughout the world, and maintain, in every country, numerous and flourishing establishments formed on the original model of Graeffenberg.

The following is a pretty full account of his *methodus medendi:*

In his first interview with the patient, after hearing sufficient to give him a rude insight into the locality and general features of the malady, Preissnitz proceeds to investigate its suitableness to his method of cure. He does this by sprinkling the surface of the body with cold water, or witnessing the taking of a cold bath, and then watching the development of reaction. If this appears in a certain amount of activity, he pronounces his case appropriate for his treatment; if not, he advises the abandonment of all hydropathic intentions. This is a mode of ascertaing the the power of the constitution quite original, and it cannot be said to be unscientific. The power of resisting the external application of cold is a most essential conservative property of the animal system, and the degree to which it exists must be regarded as, in some respects, a criterion of the amount of *vis medicatrix* possessed by the patient. We see no very decisive reason for pronouncing it a more fallacious guide than the orthodox custom of feeling the pulse. The only objection to it that occurs to us is that it may not be always free from hazard

This point being satisfactorily determined, the patient is straightway admitted into the mysteries of the cure. In the first place, he finds himself restricted to a peculiar diet. Every stimulant is absolutely prohibited, from brandy and claret, to mustard and pepper; so, also, are most of the luxuries imported from foreign parts, such as tea, coffee, and every kind of spice. The meals consist of three—breakfast at eight, dinner at one, and supper at seven or eight o'clock. For breakfast, cold milk is the beverage, and bread and butter its only substantial companions. At dinner, there is no other restriction than those above named. Supper is a repetition of breakfast, with the occasional addition of preserved fruit or potatoes. Throughout the day, no warm beverage whatever is permitted, and much of the food is brought to table considerably cooled. As some compensation for these mrnifold deprivations, the patient is allowed to gratify his appetite with every reasonable variety, and a free abundance of substantial and nutritious food. He finds it a maxim that generous diet will promote his recovery, the treatment being responsible for preventing surfeit. He no longer finds an embargo laid on fruit and vegetables, and is not expected to dine seven days in the week off dry bread and mutton chops. So that, on the whole, there is perhaps about an equal amount of indulgence and restriction, as respects diet, to a patient coming to the Graeffenberg rules from those of some fashionable physician in London.

In the next place, the majority of patients are directed to enter upon a course of water-drinking, the quantity of water varying from five or six to thirty or forty tumblers in twenty-four hours. A large portion of this is taken before breakfast, the rest at suitable periods after meals, so as not to interfere with digestion, with frequently a glass or two the last thing at night. Exercise is generally advised at the time of water-drinking, except when this accompanies some other process of treatment incompatible with it.

A third rule insisted on is, that every patieet shall take a large amount of exercise during the day. This is, to some degree, indispensable after the cold baths, as a means of procuring the necessary reaction. Walking in the open air is the mode generally selected, when possible. In case of bad weather, or lameness, other plans are contrived; such as gymnastics, sawing or chopping wood, &c. As a general rule, every patient is required to take a long walk before breakfast. It is a *vexata quæstio*, we believe, among hydropathists, as among doctors, whether the patients should rest or walk immediately after a meal; but the water-doctors generally incline to advise very gentle exercise at such times; and, we believe, properly. The well-known experiments on greyhounds, and such other convincing facts, are counterbalanced, to say the least, by the habits of the working man, who proceeds to his labor as soon as he has swallowed his dinner, and rarely suffers from doing so.

After these preliminaries, and the case being pronounced suitable for the treatment, the next morning witnesses the patient's initiation into more active preceedings. At an early hour of the morning, varying according to the time required for the operation about to be undergone, a bath attendant enters with the formidable machinery for the administration of a rubbing with a wet sheet, a packing in the dry blanket, or a packing in the wet sheet. The first of these processes consists of throwing a wet sheet over the whole person, and applying upon it active friction of a few minutes' duration. A glow is thus excited. The patient then dresses, takes his water, and sets forth upon his morning's walk. The second of the above three operations requires the patient to be enveloped in several blankets, with perhaps the superincumbence of a large feather pillow, until free perspiration is excited, which generally requires a period of about three hours. When the perspiration has continued the prescribed time (from fifteen minutes to an hour, or more,) the patient is subjected to some kind of cold bath, either by the wet sheet, as just described, by pouring water over the person from pails or watering pots, or by taking a plunge bath. This being followed by friction and water drinking, the morning's proceedings are concluded by exercise. Packing in the wet sheet is similar to the foregoing, with the addition, next the skin, of a sheet wrung out of cold water, It is generally of shorter duration, as forty-five minutes or an hour, the object being to excite a glow, instead of perspiration. It is followed by cold bathing, as just described. During the packing, in both instances, some glasses of cold water are imbibed through a tube.

At other parts of the day, other portions of the treatment are applied, such as the sitz-bath, the douche, the shower-bath, head-bath, foot-bath, &c.

The sitz-bath is a tub of cold water, in which the patient sits for a period varying from a few minutes to an hour, or even longer, using constant friction to the abdominal region. The other baths mentioned in this paragraph need no description. These, as well as the former processes, are sometimes repeated during the day. In certain cases the day's proceedings commence with some of them, in place of those previously mentioned. A rubbing with the wet sheet is frequently employed before getting into bed at night.

In fever, from whatever source, the patient is enveloped in a succession of wet sheets, renewed as often as they become warm, for a period varying with the intensity of the case—say, from 30 minutes to 5 or six hours. In other similar cases, cold immersion or affusion is employed with the same view—viz: to reduce the morbid heat of the system.

The *umschalge*, or compress, is an essential and seldom-omitted part of the treatment. It is a cloth, well wetted with cold water, applied to the surface nearest the supposed seat of the disease, securely covered with a dry cloth, and changed as often as it becomes dry during the day. It is sometimes covered with a layer of oiled silk, which, by impeding evaporation, prevents the inconvenience of frequent change. This compress speedily becomes warm, and remains so until dry. It is termed a heating or stimulating bandage. In cases of superficial infiammation it is more frequently changed, so as to be kept cold, whereby its effect is just the reverse, being then a local antiphlogistic.

In other establishments the sweating has been effected by other means than the simple envelopment of Preissnitz, as by the vapor bath, or a chamber highly heated by a stove. We have heard of a temperature of 180°, and even that of 198° Fahrenheit, being employed for this purpose. The blankets used by Preissnitz are very bad conductors of caloric, therefore they cause the heat given off by the body to be accumulated around its surface, by the lengthened influence of which the sudorific action is effected. This process differs in no other manner than in degree and rapidity of effect from exposing the same surface to heat of any other origin. The animal heat, when once evolved, becomes a quality of the surrounding atmosphere. Being kept in contact with the body by blankets, it constitutes an artificial elevation of temperature, and nothing more. Therefore, in cases where active sweating is required, we can suppose no disadvantage to result from using other kinds of artificial heat, and can easily imagine advantages in a higher temperature than that attainable from animal heat alone. But we would limit this remark to dry heat. Aqueous vapor, by a well known law, impedes evaporation, and would therefore restrict the full completion of the sudorific process. For this reason it is used to prevent plants from parting with their moisture in hot-houses. For the same reason it should *not* be used when the intention is to promote the removal of moisture, or to promote perspiration.

A point uniformly insisted on by Preissnitz is, that his patients should abstain from wearing flannel next the skin. When we consider how generally the use of this article of clothing has been advised by physicians and adopted by invalids, especially in this country, we can easily conceive that strong prejudices will exist in the minds of patients against relinquishing it. Yet it appears to be almost universally discarded by hydropathists, and, as far as we have learnt, without any mischievous consequences.

Another maxim of Preissnitz is, that his patients are never to take any kind of drug. It should be remarked that, not being licensed to practice medicine, it would be illegal for him to administer drugs. So that it does not follow, from his disuse of them, that he himself would be opposed to their use *in all cases*, much less that their use is in any way *inconsistent* with his practice. His medical disciples, not being similarly restricted, so far as we can learn, usually employ drugs occasionally, though sparingly.

Such is hydropathy, in which the following points are worthy of note: First, that all stimulants from internal parts are withdrawn; and thus the system is at once delivered from some of the most fruitful causes of disease. No alcoholic or other stimulating drinks are allowed, and the nervous system, shattered by the excesses so incident to civilized society, is permitted to regain its tone. Secondly, the system of diet enforced is such as other practitioners seldom venture to prescribe. In these circumstances we find an explanation of many of the cures of hydropathy so much vaunted. The novelist, Bulwer, worn out by a life of dissipation and mental labor, having failed to regain his health amid the excitements of his city life, went to the country, submitted to the regimen of the "water-cure," and returned to London well. To him it appeared something wonderful; so marvellous, that he must forthwith give his experience to the world; but to the physician there appeared nothing wonderful about it, except that a man of reputed sense could fail to see in his altered mode of life the cause of his cure.

A third, and certainly important, principle of hydropathic treatment is, that almost all its measures are addressed to the surface. Most physicians allow, that in our practice we are too apt to neglect the skin. Hydropathy is guilty of no such neglect. Fourthly, this system enforces a method of most energetic general and local counter-irritation; and in addition to this, the use of cold water is one of the surest modes of reducing artificial excitement, as physicians have generally understood, at least since the time of Currie. Finally, the application of cold water to the body is well known to be tonic; withal, exercise is enjoined.

Do we not find in these influences the "salt" which, thus far, has saved the system of Preissnitz? Have we not in this enumeration causes enough to explain all the cures which have resulted from hydropathy, supposing them to have been as numerous as the advocates of the system pretend they have been? Admitting this, it by no means follows that hydropathy, as a system, is to be countenanced. As practiced now, it is a system of arrant quackery; but that affords no good reason why the profession should not profit by the lessons it has taught. These superstitions will arise in medicine; in one age they come as *metallic tractors;* in a more remote one they took the shape of the *royal touch;* again it was *mesmerism;* now it is *hydropathy.* We had as well look these unwelcome intruders in the face, and weigh impartially their merits, if, perchance, they be found to possess any. If they are wholly destitute of truth—if there be no soul in them at all—they will soon die and vanish out of sight, and there is no need that we should assail them. If there be any thing in them that will avail us in our contests with disease, then "why should the devil have the good tunes to himself?" Certainly, scientific physicians can make a better application of water than the ignorant water-doctors; and after all, to adopt the language of the author of the tract before us, "the difference between quacks and respectable practitioners is one, not so much of remedies used, as of skill and honesty in using them."

